# Diagnostic yield of targeted next-generation sequencing for pediatric hereditary hemolytic anemia

**DOI:** 10.1186/s12920-023-01648-y

**Published:** 2023-09-11

**Authors:** Yu Jeong Choi, Hongkyung Kim, Won Kee Ahn, Seung-Tae Lee, Jung Woo Han, Jong Rak Choi, Chuhl Joo Lyu, Seungmin Hahn, Saeam Shin

**Affiliations:** 1 Department of Laboratory Medicine, Yonsei University College of Medicine, Seoul, Republic of Korea; 2 Department of Laboratory Medicine, Chung-Ang University Gwangmyung Hospital, Chung-Ang University College of Medicine, Gwangmyung, Republic of Korea; 3 Department of Pediatric Hematology-Oncology, Yonsei Cancer Center, Severance Hospital, Yonsei University College of Medicine, 50-1 Yonsei-ro, Seodaemun-gu, Seoul, 03722 Republic of Korea; 4 Dxome, Seoul, Republic of Korea; 5 Department of Laboratory Medicine, Severance Hospital, Yonsei University College of Medicine, 50-1 Yonsei-ro, Seodaemun-gu, Seoul, 03722 Republic of Korea

**Keywords:** Hereditary hemolytic anemia, Next-generation sequencing, Mutations

## Abstract

**Background:**

Hereditary hemolytic anemia (HHA) refers to a heterogeneous group of genetic disorders that share one common feature: destruction of circulating red blood cells (RBCs). The destruction of RBCs may be due to membranopathies, enzymopathies, or hemoglobinopathies. Because these are genetic disorders, incorporation of next-generation sequencing (NGS) has facilitated the diagnostic process of HHA.

**Method:**

Genetic data from 29 patients with suspected hereditary anemia in a tertiary hospital were retrospectively reviewed to evaluate the efficacy of NGS on hereditary anemia diagnosis. Targeted NGS was performed with custom probes for 497 genes associated with hematologic disorders. After genomic DNA was extracted from peripheral blood, prepared libraries were hybridized with capture probes and sequenced using NextSeq 550Dx (Illumina, San Diego, CA, USA).

**Result:**

Among the 29 patients, *ANK1* variants were detected in five, four of which were pathogenic or likely pathogenic variants. *SPTB* variants were detected in six patients, five of which were classified as pathogenic or likely pathogenic variants. We detected *g6pd* pathogenic and *spta1* likely pathogenic variants in two patients and one patient, respectively. Whole-gene deletions in both *HBA1* and *HBA2* were detected in two patients, while only *HBA2* deletion was detected in one patient. One likely pathogenic variant in *PLKR* was detected in one patient, and one likely pathogenic variant in *ALAS2* was detected in another.

**Conclusion:**

Here, NGS played a critical role in definitive diagnosis in 18 out of 29 patients (62.07%) with suspected HHA. Thus, its incorporation into the diagnostic workflow is crucial.

**Supplementary Information:**

The online version contains supplementary material available at 10.1186/s12920-023-01648-y.

## Background

Hereditary hemolytic anemia (HHA) refers to a heterogeneous group of genetic disorders that share one common feature: destruction of circulating red blood cells (RBCs). The destruction of RBCs may be due to membranopathies, enzymopathies, or hemoglobinopathies [1].

Membranopathies arise from mutations in genes coding for RBC membrane proteins such as spectrin (*SPTA1* and *SPTB*) and ankyrin 1 (*ANK1*) and account for more than half of HHAs [2]. Examples include hereditary spherocytosis (HS), hereditary elliptocytosis (HE), and hereditary stomatocytosis (HSt), among which HS is the most common [3]. Enzymopathies affect the cellular metabolism of RBCs, which consists of anaerobic glycolysis, hexose monophosphate shunt, glutathione metabolism, and nucleotide metabolism [4, 5]. Enzymes involved in these pathways, such as glucose-6-phosphate dehydrogenase (*G6PD*) and pyruvate kinase (*PKLR*), are the most common enzyme disorders in HHAs [6]. Hemoglobinopathies include thalassemias (abnormal hemoglobin proportion) and structurally abnormal hemoglobin. Abnormal hemoglobin molecules produced in these conditions alter the RBC membrane, leading to cell destruction [7].

HHA prevalence is low in Korea because HS is less frequent in Asians compared with Caucasians, and Korea is not located in the thalassemia belt [8]. However, prevalence has increased over time due to the increased number of immigrants from Southeast Asia through international marriages and the growing awareness of the disease entity [9–12].

Moreover, no high-sensitivity, high-specificity test was available in the past; to rule out all the possible causes of HHA-like symptoms, patients had to go through an extensive battery of tests including osmotic fragility test, flow cytometry, mass spectrometry, direct sequencing, and multiplex ligation-dependent probe amplification (MLPA). Not only was this way of diagnosing cost-intensive but not possible in most facilities because of lack of required equipment and highly trained personnel. Next-generation sequencing (NGS) can sequence multiple genes simultaneously and can offer information about copy number variations as well. Therefore, incorporation of NGS has greatly facilitated the HHA diagnostic process. In this study, we report data for patients who underwent hemolytic anemia panel by NGS at a single tertiary hospital.

## Methods

### Patients

The study included 29 unrelated pediatric and young adult patients (age ≤ 25 years) who underwent NGS testing at our center for suspected hereditary anemia from March 2017 to December 2022. Clinical information and laboratory test results, including NGS, were retrospectively obtained from electronic medical records. The primary diagnosis at the time of NGS referral was regarded as the “diagnosis at referral”. In contrast, the main diagnosis after NGS test results was denoted as the “final diagnosis”.

Research involving human specimens complied with all relevant national regulations, institutional policies and the tenets of the Helsinki Declaration (as revised in 2013). This study was approved, and the requirement for informed consent was waived by the Institutional Review Board of Severance Hospital, Seoul, Republic of Korea (4-2023-0138).

### Next-generation sequencing analysis

Genomic DNA was extracted from peripheral blood using a QIAsymphony DNA Mini Kit (Qiagen, Hilden, Germany). A custom capture panel (Dxome, Seoul, Republic of Korea) targeting coding exons and intron-exon boundaries of 497 genes related to hematologic disorders (Supplementary Table S1) was used. Prepared libraries were hybridized with capture probes and sequenced as paired-end reads (2 × 150 bp) using NextSeq 550Dx (Illumina, San Diego, CA, USA). NGS data analysis was performed with DxSeq Analyzer (Dxome). Single-nucleotide variants, small insertion and deletions, and copy number variants were identified [13, 14]. All variants were classified and reported as a 5-tier system according to the American College of Medical Genetics and Genomics and the Association for Molecular Pathology guidelines [15]. Genes included in our panel that are associated with HHAs are classified according to their relevant phenotypes in Table 1.

**Table 1:** Genes included in our panel classified according to phenotype

Phenotype	Genes included in the panel
Membranopathy	*ANK1, SPTB, SPTA1, SLC4A1, EPB42, EPB41, PIEZO1, RHAG*
Enzymopathy	*G6PD, PKLR, AK1, AK2, ALAS2, GPI, NT5C3A, GCLC, GPX1, GSR, GSS, HK1, BPGM, TPI1, PFKL, PFKM, PGK1, ALDOA*
Hemoglobinopathy	*HBB, HBA1, HBA2, HBD*

### Statistical analysis

Microsoft Excel 2013 (Seattle, WA, USA), Statistical Package for the Social Sciences (SPSS) v.23 (SPSS Inc., Chicago, IL, USA) were used for statistical analyses. Continuous variables were evaluated for normality using the Shapiro-Wilk test. The Mann-Whitney U test or independent two-sample t-test was employed according to the normality of independent variables. Fisher’s exact test was used to compare categorical variables. All parameters without normal distributions were presented as median with first and third quartiles. A *P*-value < 0.05 was interpreted as being statistically significant. Diagnostic yield was calculated by dividing the number of patients in whom at least one likely pathogenic or pathogenic (LP/P) variant was detected by the total number of patients. If only one LP/P variant was detected in an autosomal recessive gene, it was excluded from the calculation.

## Results

Patient demographics are summarized in Table 2. Patients were divided into those with LP/P variants versus those with only variants of uncertain significance (VOUS) or no variants (none). The LP/P group was younger; showed higher reticulocyte indices and lactate dehydrogenase (LDH) and total bilirubin levels; and was more likely to have poikilocytosis. However, no statistically significant difference was observed between the two groups in terms of age, sex, hemoglobin level, reticulocyte count, red cell distribution width (RDW), LDH, and total bilirubin.

**Table 2:** Patient demographics

Characteristics		LP/P	VOUS/none	Total	*P*-value
Age (yrs)					0.084
	N	18	11	29	
	Median	5	14	8	
	IQR	6.75	18	11.5	
Gender, n (%)					0.125
	Female	9 (50.0)	9 (81.8)	18 (62.1)	
	Male	9 (50.0)	2 (18.2)	11 (37.9)	
Hb (g/dL)					0.794
	N	18	11	29	
	Mean (SD)	10.03 (1.99)	10.25 (2.28)	10.11 (2.11)	
Reticulocyte (%)					0.146
	N	17	8	25	
	Mean (SD)	9.35 (4.54)	6.07 (6.16)	8.57 (5.15)	
Reticulocyte count (×10^3^/µL)					0.117
	N	17	8	25	
	Mean (SD)	323.44 (158.19)	203.35 (200.29)	291.94 (178.26)	
RDW (%)					0.233
	N	18	11	29	
	Mean (SD)	18.77 (3.30)	17.16 (3.67)	18.16 (3.50)	
Poikilocytosis					0.044
	-	4	7	11	
	1+	8	1	9	
	2+	1	2	3	
	3+	1	0	1	
	4+	1	0	1	
LDH (IU/L)					0.872
	N	18	8	26	
	Mean (SD)	348.72 (165.63)	337.75 (154.53)	345.35 (135.33)	
Total bilirubin (mg/dL)^*^					0.106
	N	17	9	26	
	Mean (SD)	2.69 (2.15)	1.33 (1.48)	2.22 (2.03)	

^*^Two values below the analytical measurement range (< 0.15) were excluded

Abbreviations: LP, likely pathogenic; P, pathogenic; VOUS, variant of unknown significance; SD, standard deviation; Hb, hemoglobin; RDW, red blood cell distribution width; LDH, lactate dehydrogenase

Among the 29 patients, *ANK1* variants were detected in five, four of which were LP/P variants (Table 3). *SPTB* variants were found in six patients, five of which were classified as LP/P variants. Of three variants identified in the *SPTA1* gene, one was classified as LP, while the other two were classified as VOUS. VOUS variants in the *SLC4A1* gene were found in two cases, while four VOUS and one LP variant of *PIEZO1* were detected in five patients. Pathogenic *G6PD* mutations were each identified as hemizygotes in two male patients. One likely pathogenic variant and one VOUS variant in the *PKLR* gene were detected in two patients, and one LP variant of *ALAS2* was detected in another. Other enzyme-related genes with variants included *GSR* and *GPI*, but the variant was VOUS or the patient was a heterozygote carrier. Whole-gene deletions in both *HBA1* and *HBA2* were detected in two patients while lone *HBA2* deletion was detected in one patient. One patient had a mutation at the start codon of *HBB*.

**Table 3:** List of variants detected in genes related to HHA and their ACMG classification scores

Case	Diagnosis at referral	Hemolytic anemia-related FHx	Gene	DNA	Protein	Zygosity	Pathogenicity	ACMG criteria	Final diagnosis
Case 1	Hereditary spherocytosis	None	*ANK1*	c.4585 C > T	p.Arg1529Ter	Hetero	P	PVS1, PM2, PP5	Hereditary spherocytosis
			*SPTA1*	c.5269 C > T	p.Arg1757Cys	Hetero	VOUS	PM2, PP5	
Case 2	Hereditary spherocytosis	None	*ANK1*	c.1737 C > A*	p.Tyr579Ter*	Hetero	LP	PVS1, PM2	Hereditary spherocytosis
Case 3	Hemolytic anemia	None	*ANK1*	c.2450 C > A	p.Ser817Ter	Hetero	LP	PVS1, PM2	Hereditary spherocytosis
			*GSR*	c.210 C > G	p.Ile70Met	Hetero	VOUS carrier	-	
Case 4	Hemolytic anemia	None	*ANK1*	c.3380del	p.Lys1127SerfsTer19	Hetero	LP	PVS1, PM2	Hereditary spherocytosis
Case 5	Hemolytic anemia	None	*ANK1*	c.5495 A > T*	p.Asn1832Ile*	Hetero	VOUS	-	Hemolytic anemia
Case 6	Hereditary spherocytosis	Cholecystectomy (mother)	*SPTB*	c.2163_2164dup*	p.Ser722CysfsTer8*	Hetero	LP	PVS1, PM2	Hereditary spherocytosis
Case 7	Congenital hemolytic anemia	Hemolytic anemia (father)	*SPTB*	c.149-8_154delinsTGG		Hetero	LP	PVS1, PM2	Hereditary spherocytosis
Case 8	Hereditary spherocytosis	Hereditary spherocytosis (mother)	*SPTB*	c.3916 C > T	p.Arg1306Ter	Hetero	P	PVS1, PM2, PP5	Hereditary spherocytosis
			*PIEZO1*	c.5134G > C	p.Val1712Leu	Hetero	VOUS	PM2, BP4	
Case 9	Congenital hemolytic anemia	Splenectomy and cholecystectomy (father)	*SPTB*	c.3855 + 1G > T		Hetero	LP	PVS1, PM2	Hereditary spherocytosis
			*GPI*	c.283-2 A > G*		Hetero	LP carrier	PVS1, PM2	
Case 10	Hemolytic anemia	None	*SPTB*	c.3855 + 2T > C		Hetero	LP	PVS1, PM2	Hereditary spherocytosis
Case 11	Hemolytic anemia	Cholecystectomy (grandmother)	*SPTA1*	c.83G > A	p.Arg28His	Hetero	LP	PS3, PP1, PP5	Hemolytic anemia
			*PIEZO1*	c.3198T > A*	p.Asp1066Glu*	Hetero	VOUS	PM2	
Case 12	Beta thalassemia	Anemia (cousin)	*HBA1, HBA2*	Whole gene deletion		Hetero	P		Alpha thalassemia
Case 13	Hemolytic anemia	None	*HBA1, HBA2*	Whole gene deletion		Hetero	P		Alpha thalassemia, dehydrated hereditary stomatocytosis
			*PIEZO1*	c.7367G > A	p.Arg2456His	Hetero	LP	PS3, PM2, PP3	
Case 14	Anemia	None	*HBA2*	Whole gene deletion		Hetero	LP		Anemia
Case 15	Hemolytic disease of fetus and newborn	Carrier of *PKLR* variant (mother and father)	*PKLR*	c.1618G > T	p.Gly540Ter	Hetero	LP	PVS1, PM2	Anemia pyruvate kinase deficiency
				c.1102G > T	p.Val368Phe	Hetero	VOUS	PM2, PP3	
			*SPTB*	c.1094T > G*	p.Leu365Arg*	Hetero	VOUS	PM2, PP3	
Case 16	Anemia	None	*G6PD*	c.1478G > A	p.Arg493His	Hemi	P	PS3, PM2, PS4_M, PP1, PP4, PP5	G6PD deficiency anemia
Case 17	Neutropenia	None	*G6PD*	c.563 C > T	p.Ser188Phe	Hemi	P	PS3, PP5_S	G6PD deficiency anemia
			*GSR*	c.94G > T	p.Glu32Ter	Hetero	LP carrier	PVS1, PM2	
			*PIEZO1*	c.5008G > C*	p.Gly1670Arg*	Hetero	VOUS	PM2	
				c.4499G > A	p.Arg1500Gln	Hetero	VOUS	BP4	
Case 18	Anemia	None	*ALAS2*	c.1355G > A	p.Arg452His	Hemi	LP	PS3, PM2, PP5	Sex-linked hypochromic sideroblastic anemia
			*SPTA1*	c.3668G > A	p.Arg1223Gln	Hetero	VOUS	-	
			*GPI*	c.893 C > T*	p.Ser298Leu*	Hetero	VOUS	-	
Case 19	Acquired hemolytic anemia	None	*PIEZO1*	c.3925 C > T	p.His1309Tyr	Hetero	VOUS	-	Acquired hemolytic anemia
Case 20	Hemolytic anemia	Splenectomy (mother)	*SLC4A1*	c.1476G > C*	p.Trp492Cys*	Hetero	VOUS	PM2, PP3	Hereditary spherocytosis
		Jaundice, anemia (mother, maternal grandmother, and aunt)		c.1447G > A	p.Gly483Ser	Hetero	VOUS	-	
Case 21	End stage renal disease	None	*PKLR*	c.1468 C > T	p.Arg490Trp	Hetero	VOUS	-	End stage renal disease
Case 22	Beta thalassemia	None	*SLC4A1*	c.1310T > C*	p.Val437Ala*	Hetero	VOUS	PM2, PP3	Anemia
Case 23	Anemia, iron deficiency	Anemia (mother, maternal grandfather)	*HBB*	c.2T > G	p.Met1?	Hetero	P	PS3, PM2, PP5	Hemolytic anemia

*Novel variants not previously reported

Abbreviations: FHx, family history; P, pathogenic; VOUS, variant of unknown significance; LP, likely pathogenic

## Discussion

In our study, NGS served as a critical diagnostic tool in 18 of 29 patients with suspected HHA (diagnostic yield: 62.07%). This LP/P variant detection rate was similar to that in previous studies using targeted NGS (54% by Fermo et al. [16], 64.9% by Russo et al. [17], and 67.3% by Nieto et al. [18], and 64.29% by Kim et al. [19] (Table 4)). Poikilocytosis was the only significantly different demographic factor between the LP/P and VOUS/none groups (Table 1). Although not statistically significant, the group with LP/P variants tended to be younger than the VOUS/none group. Newborns included due to hemolytic disease of the fetus and newborn (HDFN) included in the VOUS/none group might have masked some of the difference. Also, reticulocyte indices were noticeably elevated in the LP/P group compared with the VOUS/none group despite their differences not being significant.

**Table 4:** Summary of the diagnostic yield as well as proportion of various types of hereditary anemias in different studies

(patients)	Diagnostic yield	Membranopathy	Enzymopathy	Hemoglobinopathy	Non-hemolytic hereditary anemia
Fermo et al. [16]^a^	52.5% (64/122 patients) 54.3% (57/105 families)	67.2% (43/64)	25% (16/64)	N/A^*^	10.9% (7/64)
Russo et al. [17]^b^	64.7% (48/74)	62.5% (30/48)	25% (12/48)	N/A^*^	20.8% (10/48)
Nieto et al. [18]^c^	67.3% (111/165)	67.6% (75/111)	32.4% (36/111)	N/A^*^	1.8% (2/111)
Kim et al. [19]	71.4% (10/14)	50% (5/10)^d^	10% (1/10)^e^	40% (4/10)	0% (0/10)
Current study	62.1% (18/29)	61.1% (11/18)	16.7% (3/18)	22.2% (4/18)	5.6% (1/18)

^a^Two patients were diagnosed with both membranopathy and enzymopathy

^b^Four patients were diagnosed with both membranopathy and non-hemolytic hereditary anemia

^c^Four patients were diagnosed with both membranopathy and enzymopathy

^d^One patient only had a VOUS mutation

^e^This patient was a heterozygote carrier

^*^Hemoglobin genes were not included in the NGS panels used for these studies

Through the NGS HHA panel test, LP/P variants in membranopathy genes (*ANK1*, *SPTB*, *SPTA1*, *PIEZO1*, and *SLC4A1*) were found in 11 patients, accounting for 61.11% of the group. This finding was in line with the previous report that membranopathies accounted for more than half of HHAs [2] and other studies reported a similar proportion of membranopathies as well (Table 4). LP/P variants in enzymopathy genes, which include *G6PD, PKLR, ALAS2, GPI*, and *GSR*, were found in four cases (22.22% of confirmed HHA patients). Among these, *G6PD* and *PKLR* were the most common, which was also consistent with prior findings [6]. Last, whole-gene deletion of *HBA1* and *HBA2* and loss of function variants in *HBB* were found in 22.22% (4/18) of HHA patients.

A family history of hemolytic anemia or a history suggestive of hemolytic anemia such as cholecystectomy and splenectomy were found in seven patients with LP/P variants and one patient with two VOUS variants on *SLC4A1* (Case 20). The parents of case 15 were asymptomatic carriers of *PKLR* gene variants. A more thorough history-taking that included specific questions about whether a relative had undergone either cholecystectomy or splenectomy would have increased the detection rate of an affected family member. Moreover, considering the low awareness of this disease entity in prior generations, it seems reasonable to assume the prevalence of a positive family history was underestimated.

Upon review, the *SPTA1* R28H variant (case 11) was reclassified because we found a functional study that demonstrated no detectable affinity of the α-spectrin harboring R28H mutation to the β-subunit [20]. Thus, with the addition of a PS3, the then VOUS variant was reclassified as LP and reported to the clinician. Another variant, *PIEZO1* R2456H (case 13), was also reclassified from VOUS to LP based on a functional study showing prolonged cation channel activity resulting in reduced osmotic fragility [21–24].

The clinical history of Case 13 is notable because it demonstrates the value of NGS in diagnosing HHA. When the patient was seven years old, his Hb dropped to 5.6 g/dL and he received a blood transfusion. Since he had no known family history of anemia, the clinician suspected leukemia and performed a bone marrow examination, with a negative result. Results of the Coomb’s test, paroxysmal nocturnal hemoglobinuria (PNH) test, G6PD enzyme activity test, osmotic fragility test and Hb electrophoresis test were all within normal limits. Two years prior to NGS testing, by the age of 21, he had undergone cholecystectomy and splenectomy due to gallbladder stone. In 2017, the patient was referred to our hospital due to hemolytic anemia. Subsequent NGS analysis uncovered a whole-gene deletion of *HBA1* and *HBA2*, alongside an LP/P variant in *PIEZO1*, leading to a conclusive diagnosis of alpha thalassemia minor combined with dehydrated hereditary stomatocytosis. While each of these diseases individually is typically associated with mild anemia and infrequent symptom manifestation, the combined effect of these genetic aberrations accounted for the severity observed in this case. Additionally, this genetic combination shed light on the absence of any notable family history pertaining to these diseases. If NGS had been available when he was seven years old, he would not have had to do all those tests, especially a bone marrow biopsy. This case is representative of the limitations of conventional HHA tests and how NGS can overcome them.

The biggest limitation of this study is the small number of patients; if we had compared larger groups of patients, we might have obtained a significant difference between the LP/P and VOUS/none groups and been able to guide clinicians on the demographic factors and biochemical results most suggestive of HHA. Also, because this was a retrospective study, some test results were missing, and samples from other potentially affected family members could not be obtained. Nevertheless, we demonstrated the value of NGS for a confirmatory diagnosis of HHA. Moreover, NGS test results can aid patients in genetic counseling and family planning. Further studies with larger HHA cohorts and genotype-phenotype correlation information are needed.

## Conclusion

NGS played a critical role in definitive diagnosis in 18 of 29 patients (62.07%) with suspected HHA because the disease comprises overlapping phenotypes and ample genetic heterogeneity, hindering an accurate diagnosis. Through our review of patient data, we demonstrated the invaluable role of NGS in HHA diagnosis and the time and resources that can be saved if such analysis is performed at an early stage of suspicion. Thus, incorporation of NGS into the diagnostic workflow is crucial.

### Electronic supplementary material

Below is the link to the electronic supplementary material.

Supplementary Material 1

## Data Availability

The datasets generated and/or analysed during the current study are available in ClinVar, SCV003932458, SCV003932459, SCV003932460, SCV003932461, SCV003932462.

## List of abbreviations

G6PD: glucose-6-phosphate dehydrogenase
Hb: hemoglobin
HDFN: hemolytic disease of the fetus and newborn
HE: hereditary elliptocytosis
HHA: hereditary hemolytic anemia
HS: hereditary spherocytosis
HSt: hereditary stomatocytosis
LDH: lactate dehydrogenase
LP: likely pathogenic
MLPA: multiple ligation-dependent probe amplification
NGS: next-generation sequencing
P: pathogenic
RBC: red blood cell
RDW: red cell distribution width
VOUS: variants of uncertain significance
