# Supplementary material for: Diagnostic yield of targeted next-generation sequencing for pediatric hereditary hemolytic anemia
Source: BMC Med Genomics. 2023 Sep 11;16:215. doi: 10.1186/s12920-023-01648-y (PMC10496260; doi:10.1186/s12920-023-01648-y)
Supplement: Supplementary file 1 — Supplementary Material 1 [file 12920_2023_1648_MOESM1_ESM.docx]

**Supplementary Table S1.** Genes included in the next-generation sequencing panel used in this study

| *ABCB1, ABCB7, ABCG2, ABCG5, ABCG8, ABL1, ABL2, ACD, ACTB, ACTN1, ADA, ADAMTS13, AIRE, AK1, AK2, AKT2, ALAS2, ALDOA, AMN, ANK1, ANKRD26, AP3B1, ARID1A, ARPC1B, ASXL1, ATG2B, ATM, ATR, ATRX, AXIN1, BCL11B, BCL2, BCL6, BCOR, BCORL1, BHLHE41, BIRC3, BLM, BPGM, BRAF, BRCA1, BRCA2, BRCC3, BRINP3, BRIP1, BTG1, BTK, BTLA, C3, C4BPA, C4BPB, CALN1, CALR, CARD11, CASP10, CBL, CBLB, CBLC, CCND1, CD200, CD247, CD27, CD36, CD3D, CD3E, CD40LG, CD46, CD58, CD59, CD79B, CDAN1, CDKN1B, CDKN2A, CDKN2B, CEBPA, CFB, CFH, CFHR1, CFHR3, CFHR4, CFHR5, CFI, CHD1, CHD4, CHD9, CHMP2B, CLPB, CNOT3, COX4I2, CREBBP, CRLF2, CSF1R, CSF2RA, CSF3R, CTC1, CTCF, CTSC, CUBN, CUX1, CXCR4, CYB5R3, CYBA, CYBB, CYCS, DCLRE1C, DDX41, DGKE, DGKH, DHFR, DIS3, DKC1, DNM2, DNMT1, DNMT3A, EBF1, ECT2L, EED, EGFR, EGLN1, EGLN2, EGLN3, EHMT1, ELANE, EP300, EPAS1, EPB41, EPB42, EPCAM, EPO, EPOR, ERCC4, ERG, ETNK1, ETV6, EZH2, F2R, FANCA, FANCB, FANCC, FANCD2, FANCE, FANCF, FANCG, FANCI, FANCL, FANCM, FAS, FASLG, FAT1, FBXW7, FCGR1A, FCGR3B, FERMT3, FLI1, FLNA, FLT3, FOXP3, G6PC3, G6PD, GATA1, GATA2, GATA3, GCLC, GFI1, GFI1B, GIF, GINS1, GLRX5, GNAS, GNB1, GP1BA, GP1BB, GP9, GPI, GPRC5A, GPX1, GSKIP, GSN, GSR, GSS, HAX1, HBA1, HBA2, HBB, HBD, HCLS1, HFE, HIF1A, HIF1AN, HIF3A, HK1, HNRNPK, HOOK1, HOXA10, HOXA11, HRAS, HSPA9, HUWE1, ID3, IDH1, IDH2, IFNG, IFNGR1, IFNGR2, IKZF1, IKZF2, IKZF3, IL12RB1, IL2RB, IL2RG, IL3RA, IL7R, IRF1, ITGA2, ITGA2B, ITGB2, ITGB3, ITK, ITPKB, JAGN1, JAK1, JAK2, JAK3, JAKMIP2, JMJD1C, KDM5C, KDM6A, KDM7A, KIF23, KIT, KLF1, KMT2A, KMT2C, KMT2D, KRAS, LAMB4, LAMTOR2, LAPTM5, LCK, LEF1, LIG4, LMNA, LMO1, LMO2, LPIN2, LRP1B, LRRC4, LUC7L2, LYL1, LYST, MAD2L2, MAGT1, MAP2K1, MAP2K2, MASTL, MBL2, MECOM, MED13, MEF2B, MEF2C, MEFV, MET, MLH1, MLLT10, MLLT3, MPL, MSH2, MSH4, MSH6, MTA1, MTAP, MTR, MTRR, MVK, MYB, MYC, MYD88, MYH9, MYSM1, NAF1, NBEAL2, NBN, NCF2, NCOR2, NF1, NHEJ1, NHP2, NLRP3, NOD2, NOP10, NOTCH1, NOTCH2, NPM1, NR3C1, NRAS, NT5C2, NT5C3A, NTRK3, NUP214, OS9, P2RY2, PALB2, PARN, PAX5, PBX1, PC, PCDHB1, PDGFRA, PDGFRB, PDHA1, PDHX, PFKL, PFKM, PGK1, PGM3, PHF6, PICALM, PIEZO1, PIGA, PIK3CD, PIK3R1, PKLR, PML, PMS2, PNP, POT1, PRDM1, PRF1, PRKACG, PRPF40B, PTCH2, PTEN, PTK2B, PTPN11, PTPN2, PTPRC, PTPRD, PUS1, RAB27A, RAC1, RAC2, RAD21, RAD50, RAD51, RAD51C, RAF1, RAG1, RAG2, RB1, RBBP6, RBM8A, RELN, RFWD3, RHAG, RHOA, RIT1, RMRP, RNF168, RPL10, RPL11, RPL15, RPL23, RPL26, RPL27, RPL31, RPL35A, RPL36, RPL5, RPS10, RPS14, RPS15, RPS17, RPS19, RPS24, RPS26, RPS27, RPS27A, RPS28, RPS29, RPS7, RTEL1, RUNX1, RUNX1T1, SAMD9L, SBDS, SBF2, SEC23B, SERPING1, SETBP1, SETD2, SF1, SF3A1, SF3B1, SH2B3, SH2D1A, SHOC2, SLC11A2, SLC19A2, SLC25A38, SLC2A1, SLC35C1, SLC37A4, SLC4A1, SLCO1B1, SLCO1B3, SLFN14, SLX4, SMARCD2, SMC1A, SMC3, SOS1, SPINK5, SPRED1, SPTA1, SPTB, SRC, SRCAP, SRP72, SRSF2, STAG1, STAG2, STAT3, STAT5B, STEAP3, STX11, STXBP2, SUZ12, SYNE1, TAL1, TAL2, TAZ, TBL1XR1, TBX1, TCF3, TCIRG1, TEC, TERC, TERF1, TERF2, TERF2IP, TERT, TET1, TET2, TET3, THBD, THPO, TINF2, TLX1, TLX3, TMPRSS6, TNFAIP3, TNFRSF13B, TNFRSF14, TNFRSF1A, TOX, TP53, TPI1, TPMT, TRAF3, TRNT1, TSLP, TSR2, TUBB1, TYK2, U2AF1, U2AF2, UBE2T, UGT1A1, UGT1A7, UNC13B, UNC13D, UNC5D, USB1, USH2A, USP9X, VHL, VPS13B, VPS45, VWF, WAS, WDR1, WIPF1, WRAP53, WT1, XBP1, XIAP, XK, XRCC2, YARS2, ZAP70, ZFHX4, ZNF197, ZRSR2, MRE11A, WHSC1, STON1, OBFC1* |
| --- |
